# Genome-wide identification and expression analysis of the *Brassica oleracea* L. chitin-binding genes and response to pathogens infections

**DOI:** 10.1007/s00425-021-03596-2

**Published:** 2021-03-19

**Authors:** Mingzhao Zhu, Shujin Lu, Mu Zhuang, Yangyong Zhang, Honghao Lv, Jialei Ji, Xilin Hou, Zhiyuan Fang, Yong Wang, Limei Yang

**Affiliations:** 1 Key Laboratory of Biology and Genetic Improvement of Horticultural Crops, Ministry of Agriculture, Institute of Vegetables and Flowers, Chinese Academy of Agricultural Sciences, Beijing, 100081 China; 2 State Key Laboratory of Crop Genetics and Germplasm Enhancement, College of Horticulture, Nanjing Agricultural University, Nanjing, Jiangsu 210095 People’s Republic of China

**Keywords:** Cabbage, Genome-wide, Chitinase family genes, Diseases

## Abstract

**Main conclusion:**

**Chitinase family genes were involved in the response of Brassica oleracea to Fusarium wilt, powdery mildew, black spot and downy mildew.**

**Abstract:**

Abstract Chitinase, a category of pathogenesis-related proteins, is believed to play an important role in defending against external stress in plants. However, a comprehensive analysis of the chitin-binding gene family has not been reported to date in cabbage (*Brassica oleracea* L.), especially regarding the roles that chitinases play in response to various diseases. In this study, a total of 20 chitinase genes were identified using a genome-wide search method. Phylogenetic analysis was employed to classify these genes into two groups. The genes were distributed unevenly across six chromosomes in cabbage, and all of them contained few introns (≤ 2). The results of collinear analysis showed that the cabbage genome contained 1–5 copies of each chitinase gene (excluding *Bol035470*) identified in *Arabidopsis*. The heatmap of the chitinase gene family showed that these genes were expressed in various tissues and organs. Two genes (*Bol023322* and *Bol041024*) were relatively highly expressed in all of the investigated tissues under normal conditions, exhibiting the expression characteristics of housekeeping genes. In addition, under four different stresses, namely, Fusarium wilt, powdery mildew, black spot and downy mildew, we detected 9, 5, 8 and 8 genes with different expression levels in different treatments, respectively. Our results may help to elucidate the roles played by chitinases in the responses of host plants to various diseases.

**Supplementary Information:**

The online version contains supplementary material available at 10.1007/s00425-021-03596-2.

## Introduction

Cabbage (*Brassica oleracea* var. *capitata* L*.*) is one of the most important leafy vegetables cultivated worldwide and is of strong economic value. However, cabbage is vulnerable to various diseases, such as Fusarium wilt (FW), powdery mildew (PM), black spot (BS), downy mildew (DM) and clubroot, during its natural growth process, which leads to large economic losses. The pathogens that cause FW, PM, BS, DM and clubroot in cabbage are *Fusarium oxysporum f. sp. Conglutinans* (Liu et al. 2017), *Erysiphe cruciferarum* (Zhao et al. 2014), *Alternaria brassicicola* (Javeria et al. 2018), *Peronospora parasitica* (Verma et al. 2018) and *Plasmodiophora brassicae* (Ji et al. 2020), respectively. In general, except for *P. brassicae*, which is a special pathogen between fungi and slime molds (Hawksworth et al. 1996), the other four pathogens are all fungi. Chitin, a polymer of *N*-acetyl-β-d-glucosamine, is widely observed in insect carapaces and the cell walls of fungi and *P. brassicae* (Merzendorfer 2011; Thornton et al. 1991). The cell wall determines the shape and strength of the pathogen cells and is a key determinant of cell morphology development. As one of the primary components of the cell wall, chitin plays a very important role in the growth and development of pathogens, as well as the defense against external stress (Kombrink et al. 2011). Chitin is also considered to be a prominent signal to induce the natural immunity of plants for the invasion of pathogens (Pentecost 2013). A previous study suggested that the perception of chitin contributes to enhanced disease resistance in certain crops, such as rice and *Arabidopsis* (Kishimoto et al. 2010; Wan et al. 2012).

Chitinase is an enzyme system that employs chitin as a substrate and hydrolyzes it to N-acetyl oligosaccharide and glucose. As a subgroup of pathogenesis-related (PR) proteins, chitinase is widely present in various organs of higher plants and can be rapidly produced and accumulated when plants are subjected to pathogen infection or abiotic stress sources, such as heavy metals and drought (Bravo et al. 2003; Wang et al. 2015; Li et al. 2018). Therefore, chitinase plays an important role in protecting plants from a variety of pathogens. The induction of pathogens can enhance the activity of chitinase in plants, which subsequently inhibits spore germination and mycelial growth and even directly degrades the chitin of the fungal cell wall (Roby et al. 1988). Ntui et al. (2011) increased tobacco resistance to FW by transferring chitinase genes into tobacco. The same results were also obtained in tomatoes (Jabeen et al. 2015). Similarly, transgenic grapes carrying the wheat chitinase gene exhibit increased resistance to DM (Nookaraju et al. 2012). Marchant et al. (1998) reported that expression of the chitinase transgene reduced the severity of BS development by 13–43% in rose. Chen et al. (2018b) found that the expression of chitinase reduced the symptoms of clubroot in Chinese cabbage. In addition, exogenous application of chitinase to barley may also inhibit the proliferation of PM pathogens.

Although the function of chitinase has been analyzed in various plant species, such as tomato (Staehelin et al. 1994), potato (Khan et al. 2017), rice (Zhao et al. 2018) and apple (Fan et al. 2015), the role of the chitinase gene family in the response of cabbage to FW, PM, BS, DM and clubroot has not been elucidated. In this study, 20 chitinase genes were identified in cabbage, and their chromosome location, gene structure, collinearity relationships, evolution and *cis-*acting regulatory elements in promoters were further analyzed. The expression patterns of the chitinase family genes in response to FW, PM, BS, DM and clubroot were also investigated. Our results may help to elucidate the role played by chitinase the responses of plants to various diseases and may establish a foundation for future research investigating the genetic improvement of cabbage.

## Materials and methods

### Genome-wide identification of the chitinase genes

The *Brassica oleracea* Genomics Database (www.ocri-genomics.org/bolbase/blast/blast.html) was employed to download the cabbage whole-genome protein sequences. The Hidden Markov Model (HMM) profile of the Chiti-bind domain was downloaded from the Pfam (http://www.sanger.ac.uk/Software/Pfam/) database and used for protein screening in HMMER 3.2.1 (*e*-value < 0.01) (Finn et al. 2011). The first part of the candidate chitinase family proteins was obtained. To obtain the second part of the candidate gene, the chitinase protein sequences of *Arabidopsis* and *Brassica rapa* were downloaded from the NCBI (https://www.ncbi.nlm.nih.gov/) as a query and submitted in a BLASTP (*P* = 0.001) search. Subsequently, the two candidate sets were combined, the redundant proteins were removed, and their conserved domains were further identified using NCBI-CDD search (https://www.ncbi.nlm.nih.gov/cdd). The subcellular locations were predicted using Cell-PLoc 2.0 (http://www.csbio.sjtu.edu.cn/bioinf/Cell-PLoc-2/).

### Construction of the phylogenetic tree

Based on the amino acid sequences of chitinase derived from cabbage, *Arabidopsis thaliana* and *B. rapa*, we used MEGA6.0 (Tamura et al. 2013) to construct an unrooted neighbor-joining phylogenetic tree (bootstrap = 1000).

### Localization analysis of the chitinase genes

We used MapInspect software to draw gene chromosome location diagrams based on information regarding chitinase genes available in the cabbage genome database (http://plants.ensembl.org/Brassica_oleracea/Info/Index).

### Collinearity analysis of chitinase genes

The microsyntenic relationships of the chitinase genes in cabbage and *Arabidopsis thaliana* were detected using BLAST with an *e*-value cutoff of 1 × 10^–5^ against the whole genomes of these species. Next, we collected the physical location of the chitinase genes on each chromosome from the respective databases. The Circos tool (Krzywinski et al. 2009) was employed to visualize the relationships between two species.

### Gene structure and conserved motif analyses

We used the MEME program (http://meme-suite.org/index.html) and NCBI-CDD (https://www.ncbi.nlm.nih.gov/Structure/cdd/wrpsb.cgi) to identify the conserved motif and protein sequences, respectively. TBtools (Chen et al. 2018a) was employed to draw the gene exon–intron structure.

### Analysis of *cis*-acting elements in chitinase genes

The *cis*-acting elements in the promoters of the chitinase genes were identified by submitting the upstream sequences (1.5 kb) of the initiation codon (ATG) of each chitinase gene to PlantCARE (http://bioinformatics.psb.ugent.be/webtools/plantcare/html).

### Plant materials and treatments

The *F. oxysporum* employed in this study belongs to race 1, which is the primary race worldwide. Inbred lines 01–20 and 96–100 utilized for inoculation are susceptible and resistant to *F. oxysporum*, respectively. The roots of seedlings with three real leaves were soaked in a 1 × 10^6^ cfu/ml spore suspension for 15 min, and the seedlings were subsequently transferred to 32-well plugs. Two leaves from each plant of 01–20 and 96–100 at 0, 3, 6 and 9 dai (day after inoculation) were collected (18 individuals per treatment, 6 individuals per replicate) for RNA extraction.

The *P. brassicae* used in this study belongs to race 4 based on the differential sets of Williams (1966). A resting spore inoculum of 2 × 10^8^ spores/ml was prepared prior to inoculation. Two commercial cabbage cultivars, Xiangan 336 and Jinfeng No. 1, which were resistant and susceptible to *P. brassicae*, respectively, were sown in 32-well (8 × 4) plugs. When the seedlings grew to two real leaves, we employed a pipette to inject 2 ml of resting spore suspension into the soil around the roots of each seedling. Two kinds of treatments were performed for each cultivar. A treatment without inoculation served as the control. Eight different root tissue samples, including Jingfeng No. 1 not inoculated at 7 days, Jingfeng No. 1 inoculated at 7 days, Xiangan 336 not inoculated at 7 days, Xiangan 336 inoculated at 7 days, Jingfeng No. 1 not inoculated at 28 days, Jingfeng No. 1 inoculated at 28 days, Xiangan 336 not inoculated at 28 days and Xiangan 336 inoculated at 28 days, were collected (24 individuals per treatment, 8 individuals per replicate) for RNA extraction.

The cabbage material used for the PM inoculation experiment was the cabbage inbred line D157. When the seedlings grew to 4–5 real leaves, a resting spore suspension of 1 × 10^5^ spores/ml was sprayed evenly onto the leaves of the plants in the treatment group. The plants of the control group were sprayed with equal amounts of sterile water. During the pod-setting period, one diseased leaf of each plant in the treatment group and one healthy leaf of each plant in the control group were taken for RNA extraction. Three replicates were employed in the treatment group and the control group, and each replicate consisted of 8 plants.

The cabbage material used for the BS inoculation experiment was cabbage inbred line W18. When the seedlings grew to 2 real leaves, a resting spore suspension of 1 × 10^4^ pfu/ml was sprayed evenly onto the leaves of the plants in the treatment group. The plants of the control group were sprayed with equal amounts of sterile water. During the heading stage, one diseased leaf from each plant in the treatment group and one healthy leaf from each plant in the control group were taken for RNA extraction. Twenty-four plants were employed in this experiment, and every eight plants were considered to be one biological repetition.

The cabbage material used for the DM inoculation experiment was the cabbage inbred line 01–20. When the seedlings grew to 2 real leaves, a resting spore suspension of 5 × 10^4^ spores/ml was sprayed evenly to the back of the leaves of the plants in the treatment group. The plants of the control group were sprayed with equal amounts of sterile water. During the heading stage, one diseased leaf from each plant in the treatment group and one healthy leaf from each plant in the control group were taken for RNA extraction. Twenty-four plants were employed in this experiment, and every eight plants were considered to be one biological repetition.

All of the samples were quickly frozen in liquid nitrogen and stored at − 80 °C until RNA extraction.

The inbred line 01–20 was introduced to China from Canada in 1966 by the Institute of Vegetables and Flowers, Chinese Academy of Agricultural Sciences (IVF-CAAS). Also, 96–100, D157 and W18 are backbone inbred lines cultivated by the cabbage-broccoli research group of IVF-CAAS for many years. Jinfeng No. 1 was developed by China Vegetable Seed Co., Ltd., and Xiangan 336 was developed by Syngenta Seeds. The resistance of 01–20 and 96–100 to FW has been observed by previous researchers (Lv et al. 2014). Similarly, the resistance of Xiangan 336 and Jinfeng No. 1 to clubroot has been reported previously (Ning et al. 2018). The resistance of D157, W18 and 01–20 to PM, BS and DM, respectively, has been identified by researchers from the cabbage-broccoli research group of IVF-CAAS. The voucher specimens of all the above materials have been deposited in a public herbarium in IVF-CAAS.

### Total RNA extraction

Total RNA was extracted from cabbage samples using TRIzol following the supplier’s instructions (Transgen, Beijing, China). Then, the RNA quality was assessed using a Nanodrop spectrophotometer (Thermo Fisher Scientific, USA) and 1% formaldehyde gel electrophoresis. The cDNA was reverse transcribed with the HiScript^®^ III 1st Strand cDNA Synthesis Kit (Vazyme, Nanjing, China).

The specific primers for chitinase genes were designed with Premier 3.0 (Table S4). qRT-PCR was carried out using 2X RealStar Green Fast Mixture (GeneStar) in a Bio-Rad CFX96 Real Time PCR System. Each amplification reaction was conducted in a 20-μl reaction volume containing 10 μl KAPA SYBR, 0.5 μl of each primer, 2 μl diluted cDNA and 7 μl ddH_2_O. The PCR program was set as follows: 95 °C for 2 min followed by 40 cycles of 95 °C for 15 s, 60 °C for 30 s and 72 °C for 30 s. Melting curve analysis was performed from 65 °C to 95 °C with increments of 0.5 °C every 5 s. Three independent biological and technical replicates were performed for each reaction. The housekeeping gene actin was employed as the internal reference gene.

### Subcellular localization

The pBWA(V)HS-GLosgfp vector was used for the subcellular localization test and digested with one restriction endonucleases (BsaI) to insert the target genes. The CDS sequences of *Bol040748* were amplified with specific primer pairs with homologous arms (F: 5′-cgatGGTCTCacaacatgttcatccacaaggacaatactgcttgtccagcaaatggttt-3′; R: 5′-cagtGGTCTCatacaagcgaagggcctctgattttcacagtccaaattaggcccagttc-3′). The amplification products were recovered using the FastPure Gel DNA Extraction Mini Kit (Vazyme Biotech) and inserted into the pBWA(V)HS-GLosgfp vector, resulting in an N-terminal fusion with GFP under the control of the constitutive CaMV35S promoter. The recombinant plasmids were transferred into Agrobacterium tumefaciens strain GV3101. The fusion constructs were introduced into *Nicotiana benthamiana* protoplasts as previously described (Liu et al. 2020). The fluorescence signals were detected using confocal laser-scanning microscopy C2-ER (Nikon, Tokyo, Japan).

### Genes information and data analysis

The GenBank accession numbers of genes in *Arabidopsis* were listed in Table S1 and the detailed information about the genes in *B. oleracea* and *B. rapa* in this study can be queried through the BRADV3.0 (http://39.100.233.196/#/GeneSequence/).

All samples used for RNA sequencing and qRT-PCR in this study were set with three biological replicates. To test the repeatability among samples, all RNA sequencing data were performed principal component analysis (PCA) by RNA-Seq by Expectation Maximization (RSEM) (http://deweylab.biostat.wisc.edu/rsem/), which used transcripts per million (TPM) as the expression index. The error values of qRT-PCR data were calculated with SPSS Statistics 20.0 (SPSS, Chicago, IL, USA).

## Results

### Genome-wide identification and phylogenetic analysis of chitinase genes in cabbage

To identify chitin-binding genes in the cabbage genome (02–12), a hidden Markov model was employed to predict chitin recognition proteins in cabbage protein sequences. A total of 20 chitinase genes were identified (Table 1). The lengths of these 20 chitinase proteins ranged from 117 (*Bol030012*) to 447 (*Bol007321*) amino acids (aa). Within these 20 chitinase proteins, 13 members shared a similar localization to vacuoles, 1 to extracellular vesicles and 6 to more than one compartment.

**Table 1:** Information on cabbage chitinase genes

Gene ID	Chr	Genomic location	Gene length (bp)	Protein length (aa)	Predicted localization
*Bol007321*	C01	36002654-36007105	4452	447	Vacuole
*Bol007323*	C01	35993587-35994336	750	249	Vacuole
*Bol004604*	C03	8091984-8093153	1170	269	Extracellular vesicle/vacuole
*Bol029467*	C03	12670792-12678156	7365	408	Vacuole
*Bol029469*	C03	12681165-12682265	1101	273	Vacuole
*Bol029470*	C03	12701848-12702306	459	152	Cell wall/vacuole
*Bol035464*	C03	20687053-20689765	2713	393	Vacuole
*Bol035467*	C03	20710123-20712350	2228	340	Vacuole
*Bol035470*	C03	20721329-20722591	1263	244	Vacuole
*Bol039802*	C03	8643798-8645535	1738	262	Extracellular vesicle/vacuole
*Bol021626*	C04	39859234-39859620	387	128	Cell wall/vacuole
*Bol021627*	C04	39860449-39861636	1188	281	Vacuole
*Bol030012*	C04	1604570-1604999	430	117	Cell wall/vacuole
*Bol030015*	C04	1617304-1618422	1119	274	Extracellular vesicle/Vacuole
*Bol010293*	C05	30176713-30178044	1332	322	Vacuole
*Bol040748*	C05	494246-495037	792	231	Vacuole
*Bol041024*	C05	2018512-2020042	1531	321	Vacuole
*Bol023322*	C08	2077858-2079450	1593	322	Vacuole
*Bol025197*	C08	28963242-28964148	907	273	Vacuole
*Bol011420*	C09	680349-681621	1273	247	Extracellular vesicle

**Fig. 1:**
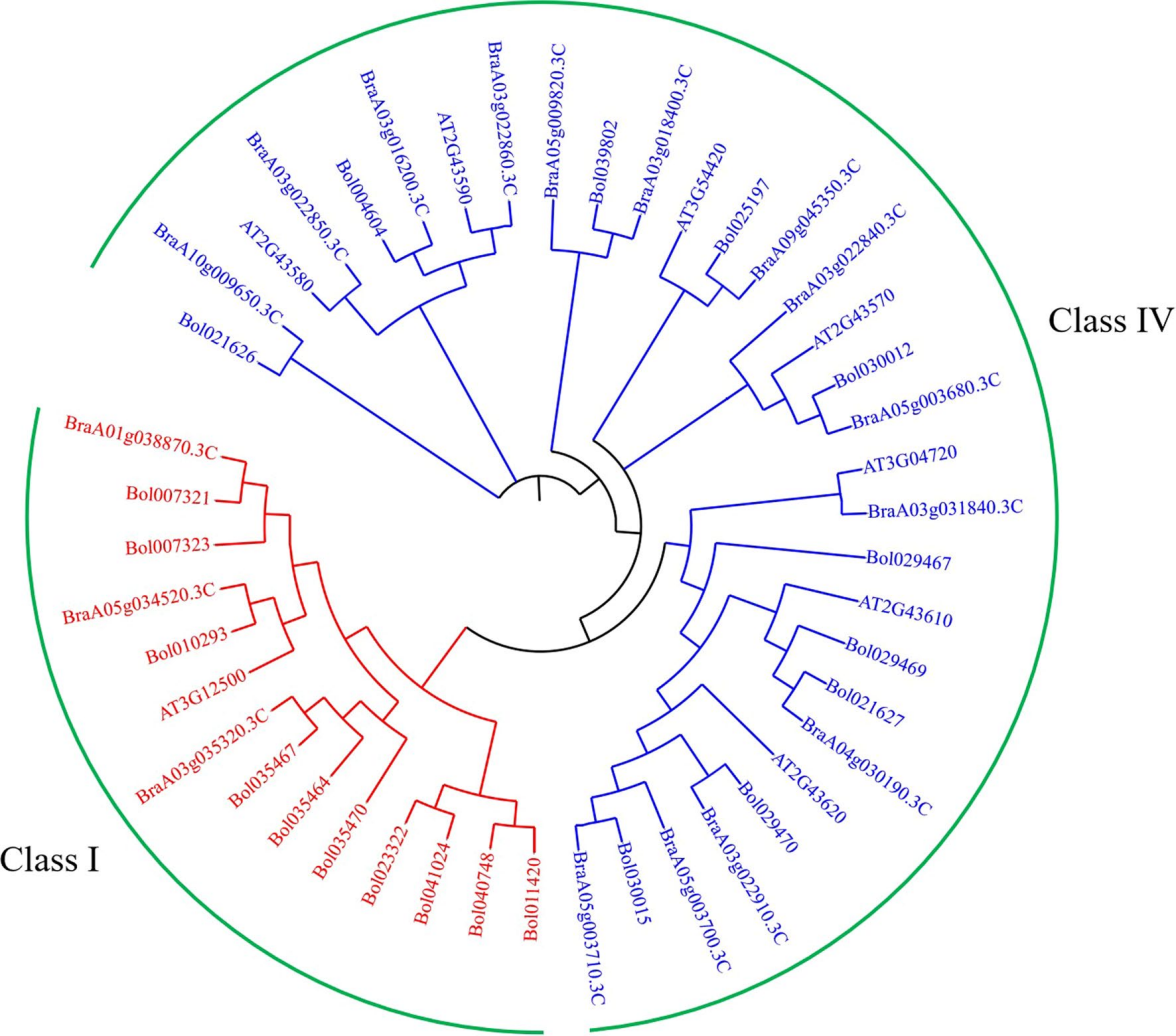
Phylogenetic tree of chitinase genes from cabbage, *A. thaliana* and *B. rapa*. The phylogenetic tree was built using the neighbor-joining (NJ) method with 1000 bootstrap replications. Roman numerals (I and IV) represent each gene cluster, which are labeled with different colors

Chitinases are classified into seven classes (Class I–VII), and most of them belong to the first four classes (Neuhaus et al. 1996). In this study, based on the amino acid sequences of the cabbage (20), *Arabidopsis* (8), and *Brassica. rapa* (17) chitinase proteins, we constructed the chitinase protein phylogenetic tree of the chitinase family genes using MEGA 6.0 software. The chitinase genes were classified into two groups (class I and IV; Fig. 1), which contained 14 members (ten cabbage, one *Arabidopsis* and three *Brassica. rapa*), and 31 members (ten cabbage, seven *Arabidopsis* and fourteen *Brassica. rapa*), respectively. All chitinases belong to the glycoside hydrolase 19 (GH-19) family, and they all have an N-terminal chitin-binding domain and a GH-19 catalytic domain (Henrissat 1991). Coincidentally, Davis et al. (2002) reported that class I and class IV chitinase genes in pineapple were induced after inoculation of *Fusarium subglutinans* f. sp. *pini*, which may indicate the special role played by these genes in plant resistance to pathogens.

### Chromosomal distribution and collinear analysis

**Fig. 2:**
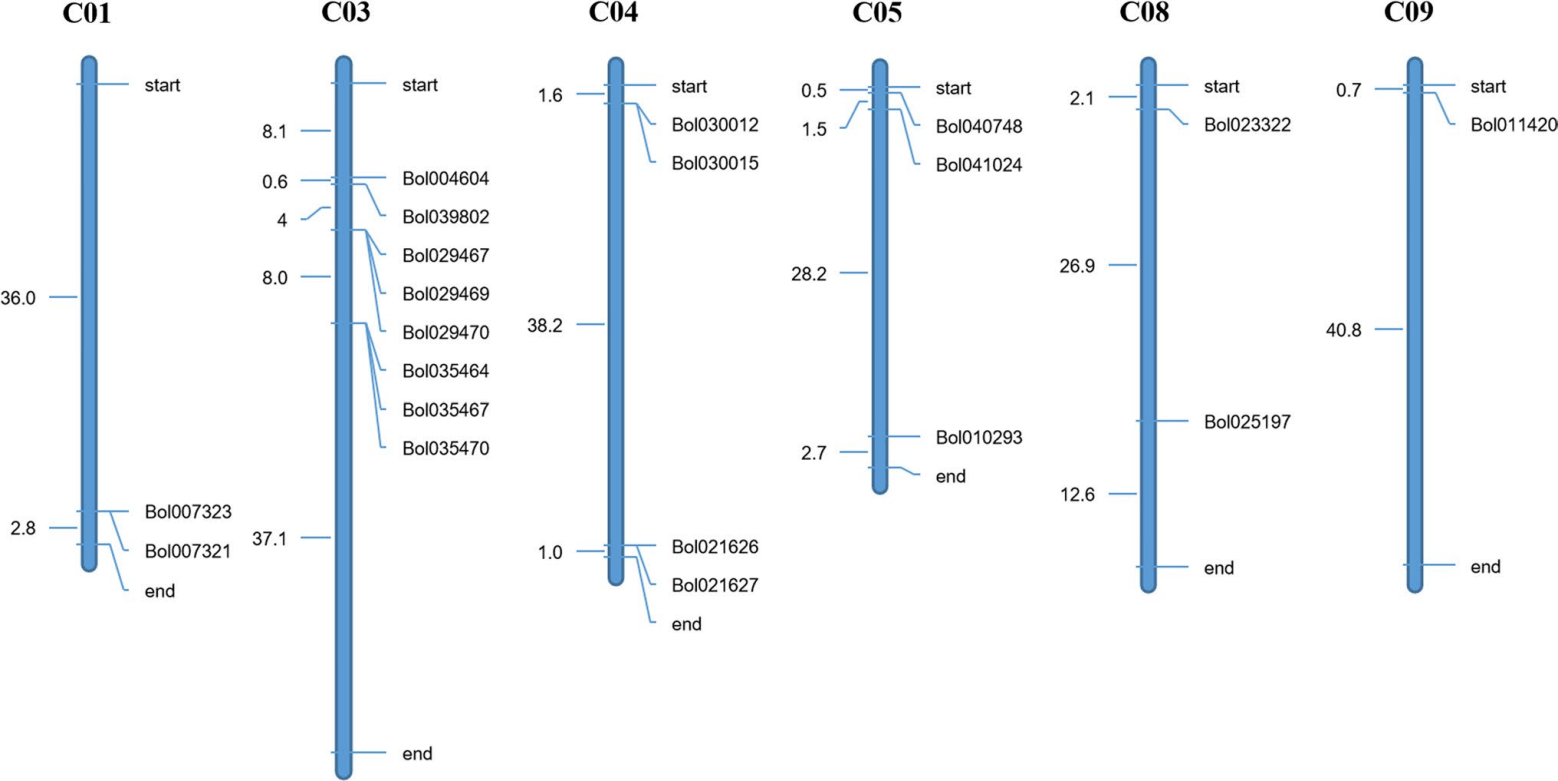
Distribution of chitinase genes in *B. oleracea.* chromosomes. The number on the top of each chromosome represents the cabbage chromosome number. Gene names are indicated on the right sides of each chromosome. The distance (Mb) between genes or genes to the ends of the chromosome is indicated on the left side of each chromosome

The 20 chitinase genes were assigned to six chromosomes of cabbage (Fig. 2). The distribution of the chitinase genes on each chromosome was uneven. The numbers of chitinase genes on each chromosome are as follows: 2 on C01, 8 on C03, 4 on C04, 3 on C05, 2 on C08, and 1 on C09.

Gene duplication is a common phenomenon in the evolution of plants, which is the reason for the formation of homologous genes in different plants. Due to the importance of gene duplications on the evolution of gene families in plants, chitinase gene replication in cabbage and the collinearity between cabbage and *Arabidopsis* of chitinase genes were analyzed.

**Fig. 3:**
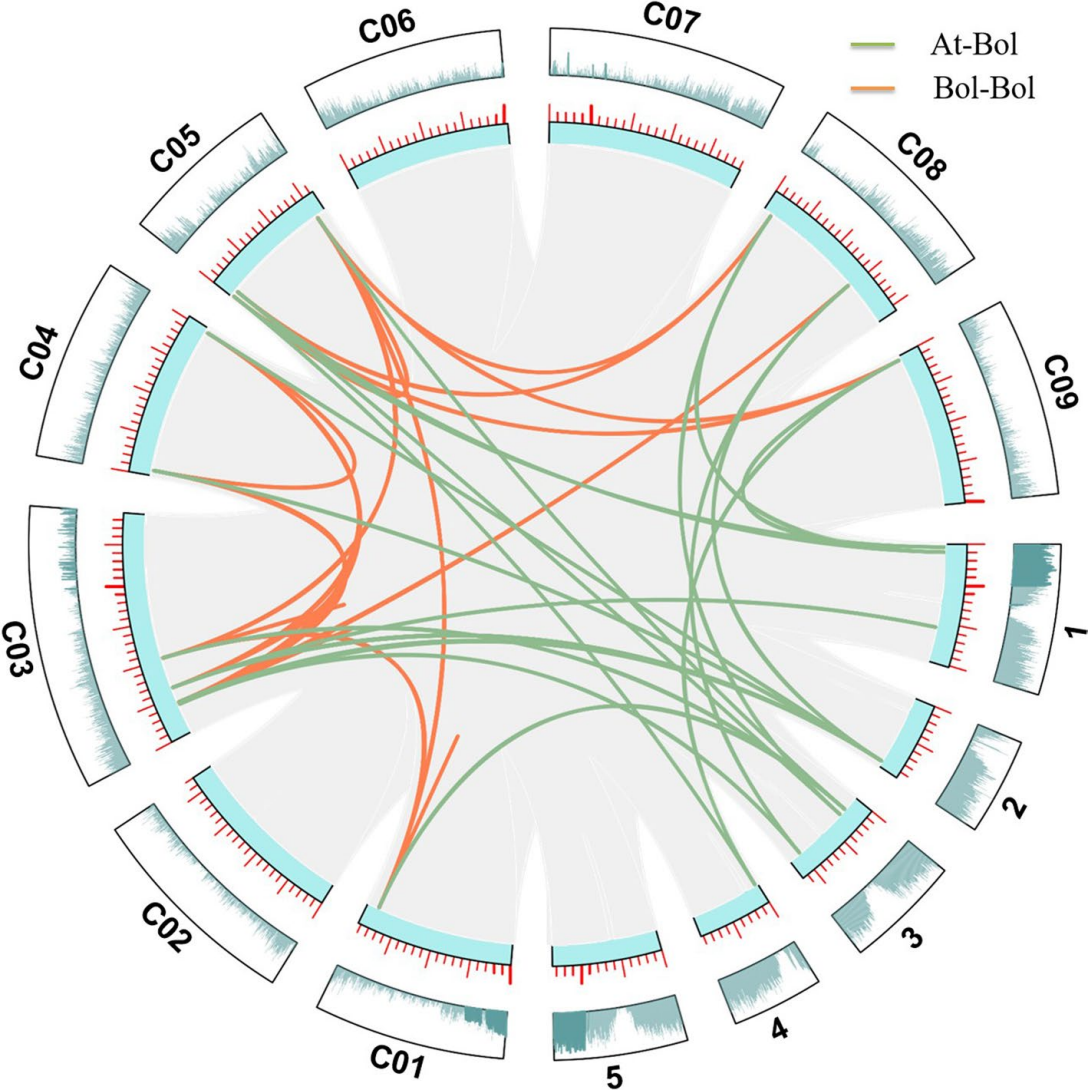
Syntenic relationship of cabbage and *A. thaliana* chitinase genes shown on the chromosome maps. C01-C09, nine chromosomes of cabbage. 1–5, five chromosomes of *A. thaliana*. Orange lines, homologous gene between cabbage chromosomes. Green lines, homologous genes between cabbage and *A. thaliana* chromosomes

The cabbage genome contained 1–5 copies of each chitinase gene (excluding *Bol035470*) found in *Arabidopsis* (Fig. 3; Table S2). For example, *AT1G56680.1* contained only one homologous gene (*Bol029467*) in cabbage, while *AT2G43590.1* contained up to five homologous genes (*Bol004604, Bol039802*, *Bol021626*, *Bol030012*, and *Bol025197*) in cabbage. In addition, 32 segmentally duplicated gene pairs were also identified among the 20 chitinase genes in the cabbage genome (Fig. 3; Table S3).

### Structure and conserved motif analysis of chitinase genes

**Fig. 4:**
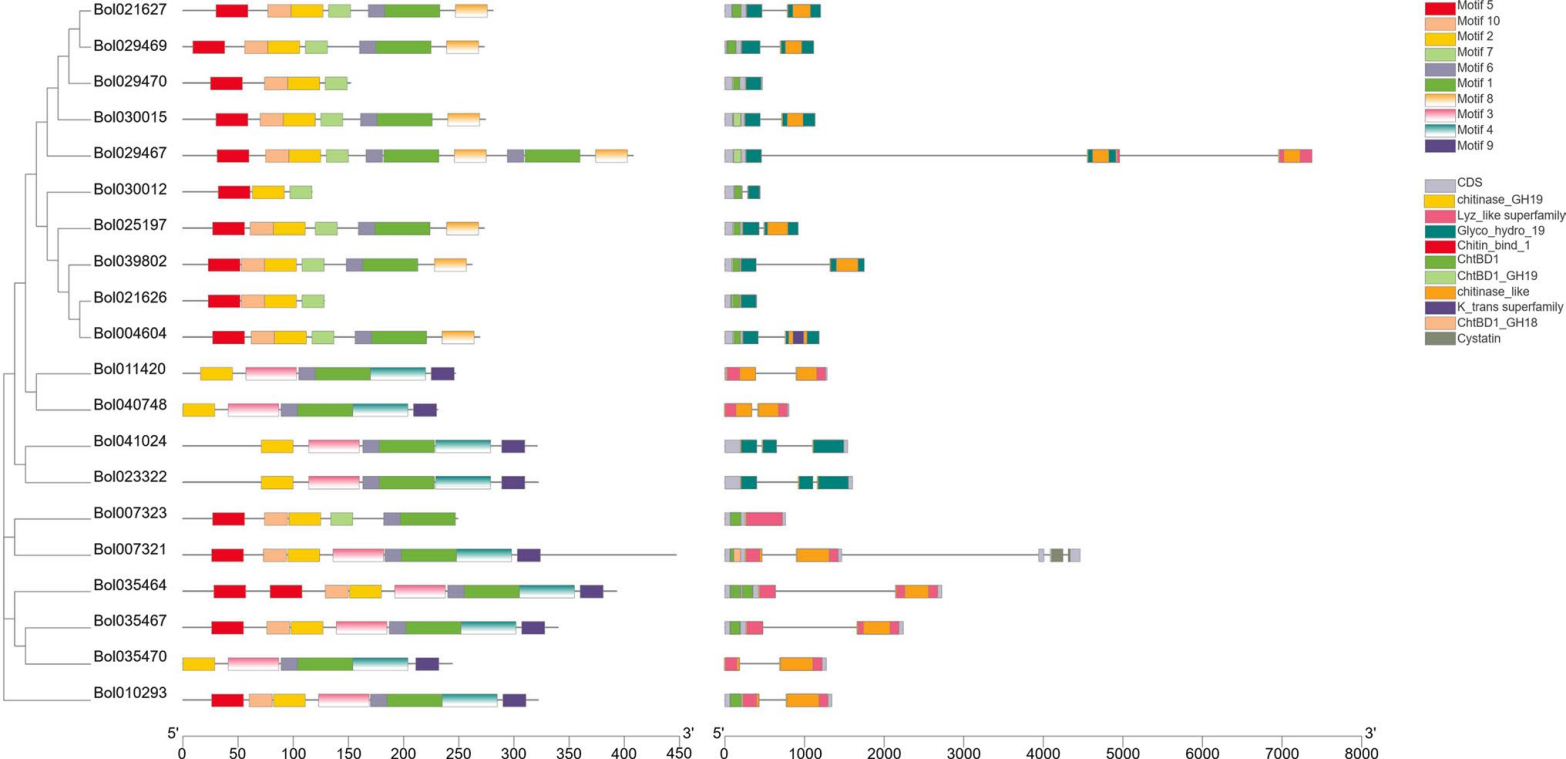
Conserved motif (**a**) and gene structure (**b**) analysis of chitinase genes. The motifs are indicated in different colored boxes. Exons are represented by boxes, while introns are represented by gray lines

To further investigate the structural diversity of chitinase genes, the gene structure among 20 chitinase genes was detected (Fig. 4b). Thirteen genes contain two exons, 4 genes contain 3 exons, and 3 genes contain only 1 exon. The lengths of exons in most genes were similar, while the lengths of introns for some genes varied widely. For example, *Bol021627* and *Bol029469* contained one shorter intron, whereas *Bol029467* contained two notably long introns.

To better understand the structural characteristics of the chitinase genes, the conserved domain and motifs were also detected (Fig. 4a, b). All members contained motifs 2 and 6. Motifs 3, 4 and 9 were uniquely present in members of Class I, while Motifs 7 and 8 were almost present in members of Class IV. Only one member of Class I contains motif 7. Among the 20 chitinase proteins, sixteen members contained motif 1, 15 members contained motif 5 and 14 members contained motif 10. Motifs 2, 3, 6, 1, 4, and 9 displayed in the same order were found in Class I, and motifs 5, 10, 2, 7, 6, 1, and 8 displayed in the same order were found in Class IV. In addition, *Bol011420*, *Bol040748*, *Bol041024, Bol023322* and *Bol035470* have the same motif composition.

As shown in Fig. 4b, most proteins contained a chitinase binding domain and GH19. Only one protein contained a K^+^ transit domain. The chitinase-like domain was present in 14 genes. The cystatin domain only existed on 1 gene (*Bol007321*). In addition, most genes in Class I contained lysozyme-like domains, which only existed on the 1 (*Bol029467*) gene in Class IV.

### The *cis*-elements in the promoters of *B. oleracea* chitinase genes

**Fig. 5:**
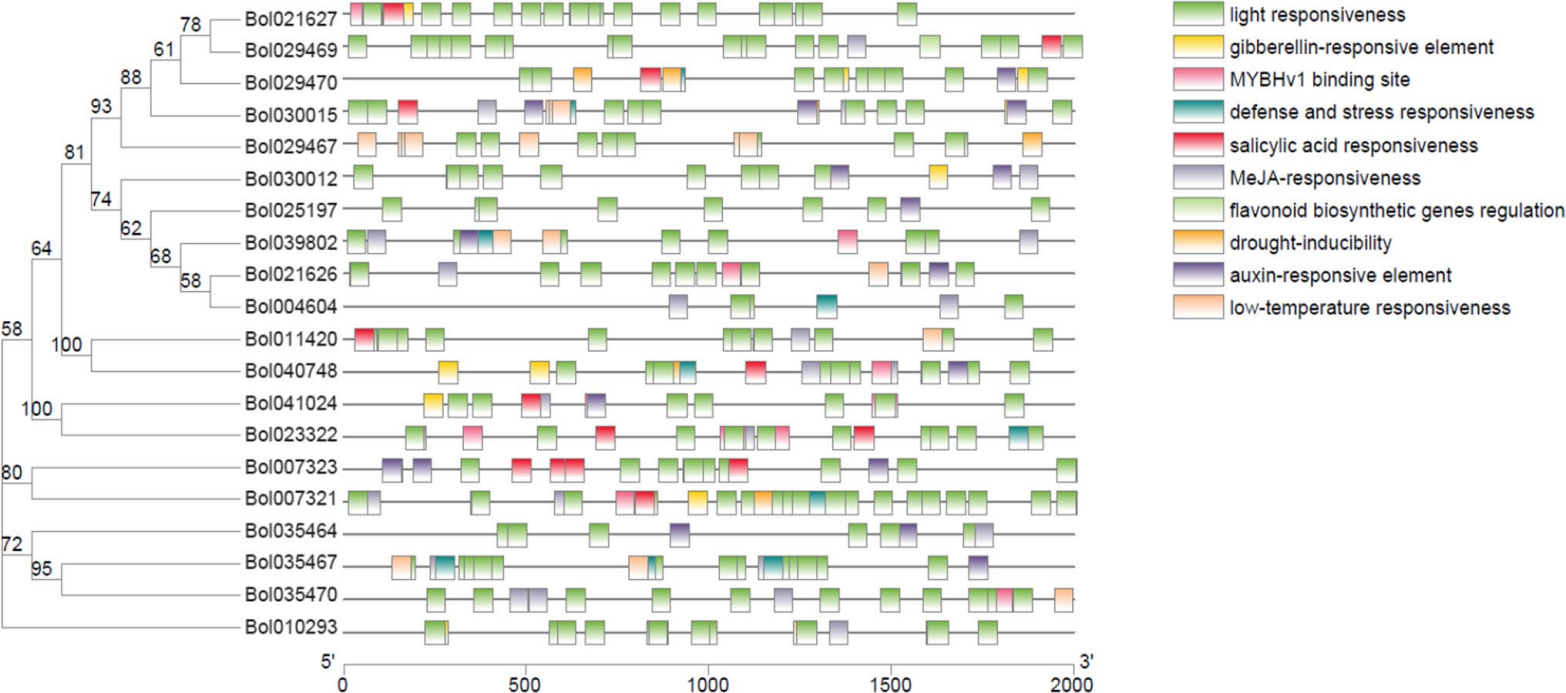
Predicted *cis*-acting elements in chitinase gene promoters

To further clarify the regulatory mechanism of chitinase genes in cabbage response to FW, clubroot, BS, PM and DM, we identified the *cis*-elements using the PlantCARE database based on the promoter sequences, and ten types of *cis*-acting regulatory elements were identified (Fig. 5). All 20 chitinase genes contained 3–17 light-responsive *cis*-elements. Seven chitinase genes contained gibberellin-responsive *cis*-elements. Eight chitinase genes contained MYBHv1-binding *cis*-elements. Salicylic acid-responsive *cis*-elements were detected in 10 chitinase genes, while *cis*-elements related to MeJA and auxin responsiveness were detected in 15 and 11 chitinase genes, respectively. In addition, the *cis*-elements related to defense and stress responsiveness, as well as low-temperature responsiveness, existed in 8 and 9 genes, respectively. The distribution of drought-inducible *cis*-elements was relatively small and was detected in only 3 genes. The *cis*-element analysis demonstrated that chitinase genes could respond to different stimuli.

### Expression patterns of chitinase genes and qRT-PCR verification

**Fig. 6:**
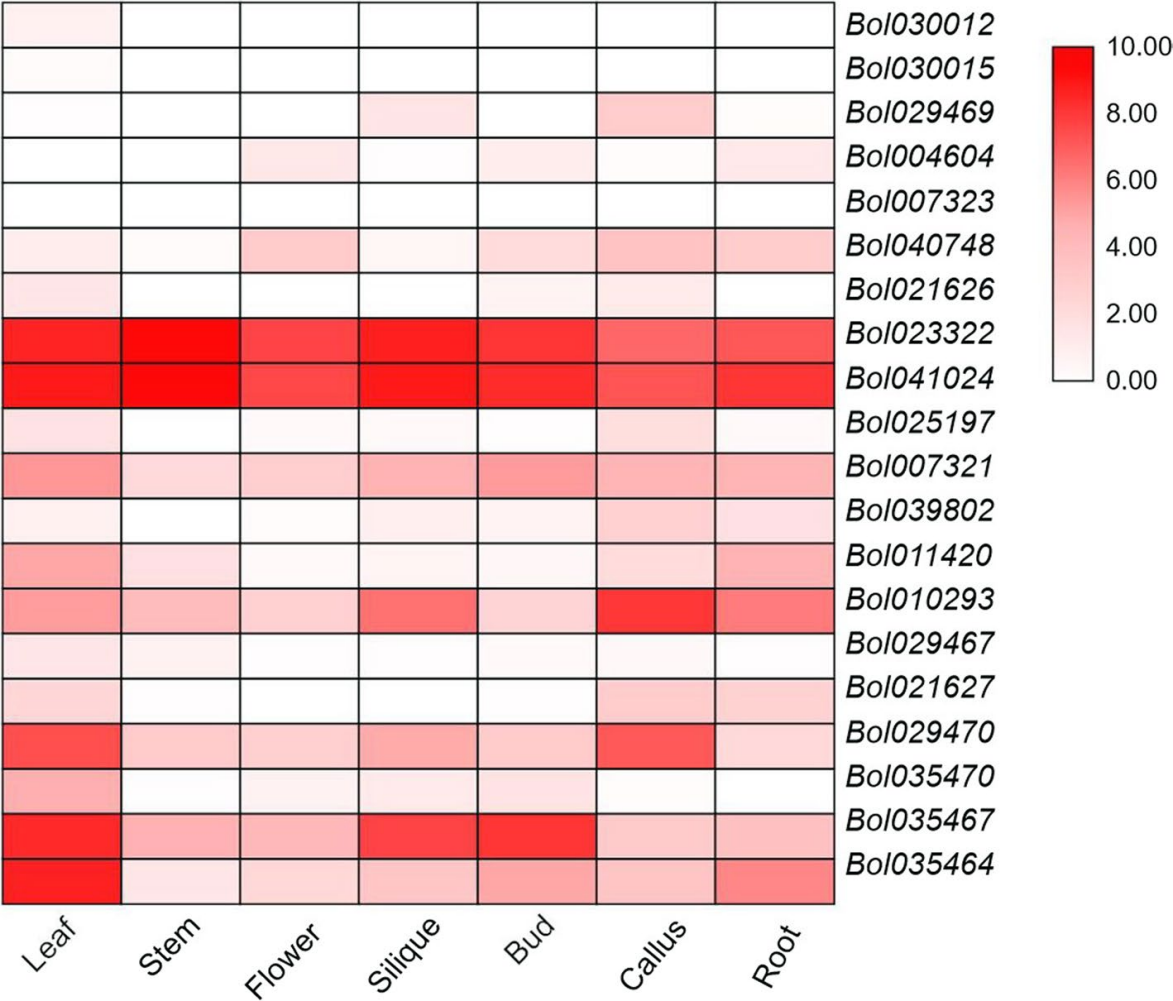
Expression of chitinase genes in different organs

The RNA-Seq dataset (GSM1052958-964) was examined to determine the expression levels of chitinase genes in the leaves, stem, flowers, siliques, buds, calli and roots of cabbage. Most of the chitinase genes exhibited different expression patterns (Fig. 6; Table S5). Eighteen of the genes were expressed in all organs, while the expression levels of two chitinase genes (*Bol030015* and *Bol007323*) were almost undetectable. Some genes were expressed only in one or two organ types, such as *Bol030012* in leaves and *Bol029469* in siliques and calluses. Conversely, *Bol023322* and *Bol041024* were highly expressed in all tissues, showing the expression characteristics of housekeeping genes (Lopes-Caitar et al. 2013). The multiple expression patterns of the chitinase genes indicate their extensive biological functions during the growth and development of cabbage.

**Fig. 7:**
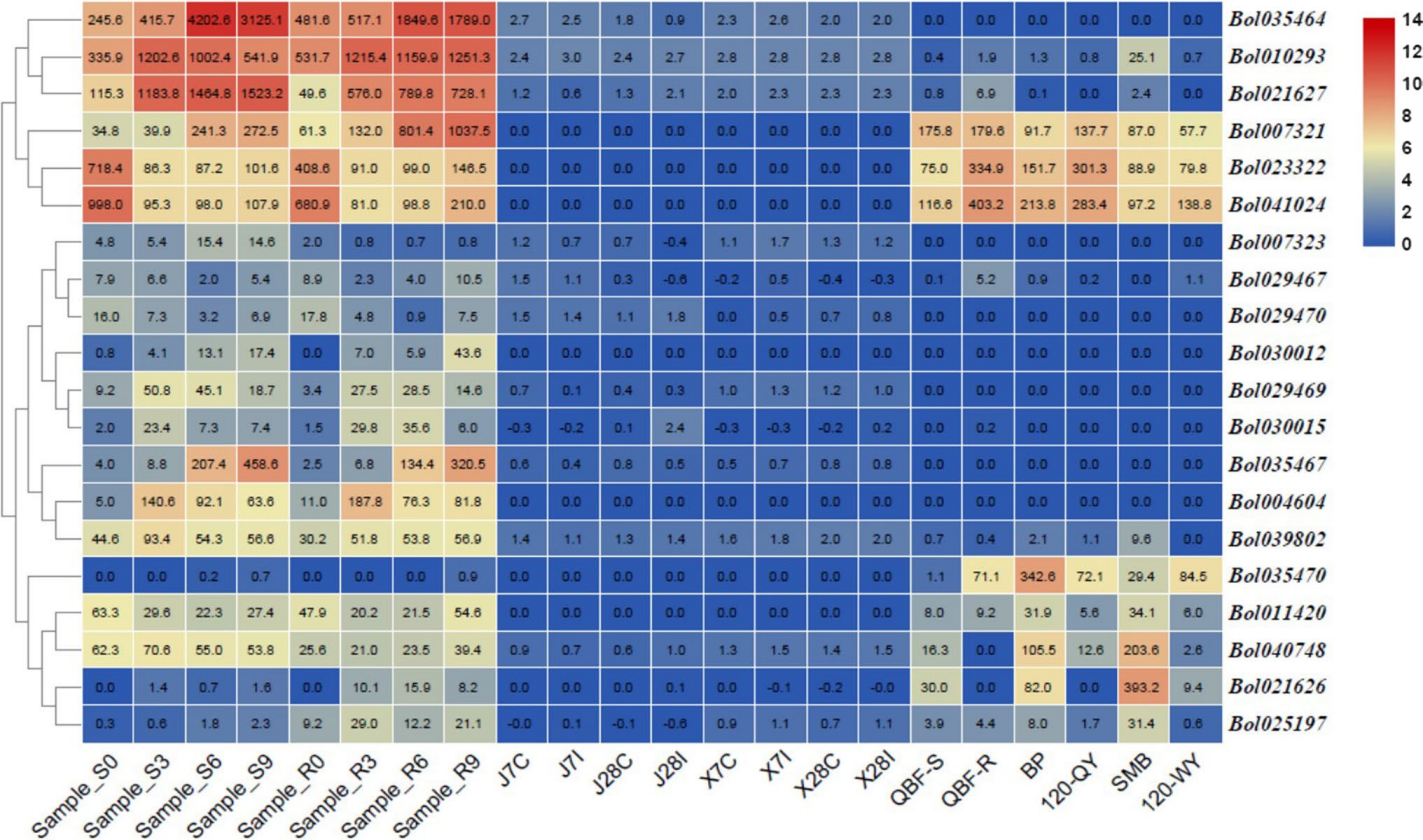
Expression patterns of chitinase genes analyzed by RNA-Seq. Samples S0, S3, S6 and S9 represented 01–20 inoculated by *F. oxysporum* at 0, 3, 6 and 9 days, respectively. Samples R0, R3, R6 and R9 represented 96–100 inoculated by *F. oxysporum* at 0, 3, 6 and 9 days, respectively. J7C, Jingfeng No. 1 not inoculated by *P. brassicae* at 7 days. J7I, Jingfeng No. 1 inoculated by *P. brassicae* at 7 days. X7C, Xiangan 336 not inoculated by *P. brassicae* at 7 days. X7I, Xiangan 336 inoculated by *P. brassicae* at 7 days. J28C, Jingfeng No. 1 not inoculated by *P. brassicae* at 28 days. J28I, Jingfeng No. 1 inoculated by *P. brassicae* at 28 days. X28C, Xiangan 336 not inoculated by *P. brassicae* at 28 days. X28I, Xiangan 336 inoculated by *P. brassicae* at 28 days. QBF-S, leaves with PM of D157 during the pod-setting stage. QBF-R, normal leaves of D157 during the pod-setting stage. BP, leaves with BS of W18 during the heading stage. 120-QY, normal leaves of W18 during heading storage. SMB, leaves with DM of 01–20 during heading storage. 120-WY, normal leaves of 01–20 during heading storage

To investigate the role played by chitinase in the response of cabbage to infection by various pathogens, we inoculated different cabbage materials with five pathogens and extracted plant tissue RNA at a specific period for transcriptome sequencing. Then, two heatmaps were established according to the RNA-seq data (Figs. 6, 7; Table S6).

**Fig. 8:**
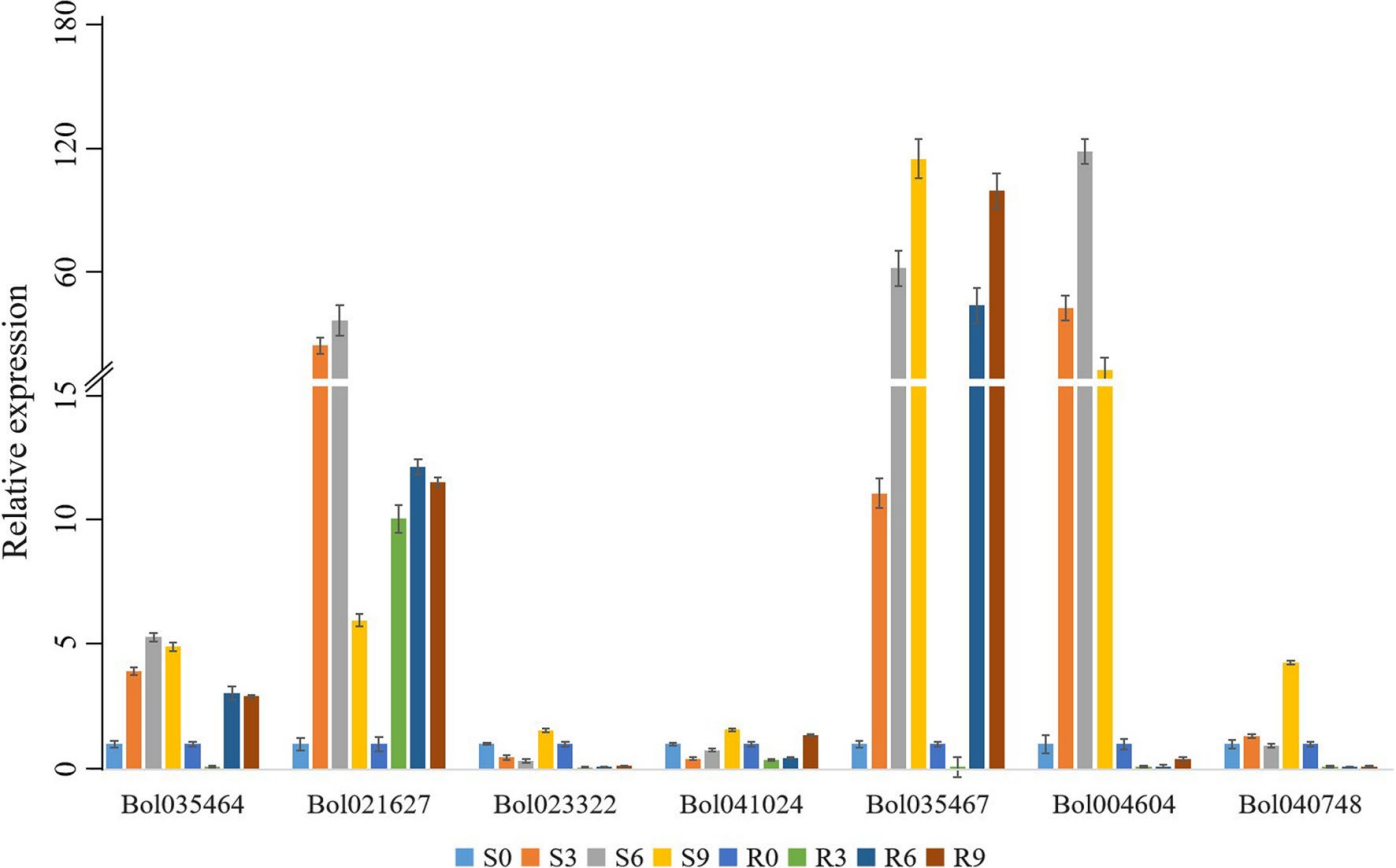
Expression levels of chitinase genes under *F. oxysporum* stress. Data are presented as the means ± SD

The expression levels of chitinase genes in 01–20 and 96–100 infected by *F. oxysporum* were notably different. Four genes (*Bol010293*, *Bol007321*, *Bol021626* and *Bol025197*) were upregulated in 96–100 compared with 01–20, while five genes (*Bol035464*, *Bol021627*, *Bol035467*, *Bol029469* and *Bol040748*) were downregulated. In different stages, the expression patterns of chitinase genes are also different. Five genes (*Bol035464*, *Bol010293*, *Bol021627*, *Bol007321* and *Bol035467*) and two genes (*Bol023322* and *Bol041024*) were significantly up- and downregulated, respectively, in both 01–20 and 96–100 after inoculation by *F. oxysporum*. The expression levels of the three genes (*Bol029469*, *Bol030015* and *Bol004604*) increased first and then decreased in both 01–20 and 96–100 after inoculation. In contrast, the expression levels of the two genes (*Bol011420* and *Bol040748*) decreased first and then increased in 96–100 after inoculation. Taken together, compared with 01–20, five genes (*Bol035464*, *Bol010293*, *Bol021627*, *Bol035467* and *Bol040748*) were significantly downregulated, and five genes (*Bol007321*, *Bol041024*, *Bol0304604*, *Bol021626* and *Bol025197*) genes were significantly upregulated, in 96–100 after inoculation. qRT-PCR was performed to verify the chitinase gene expression patterns under *F. oxysporum* stress in different inoculation periods of 01–20 and 96–100. As shown in Fig. 8, the seven genes that we detected by qRT-PCR were approximately in keeping with the results of the RNA-seq analysis, except for *Bol004604*, which further confirmed their expression patterns.

The expression patterns exhibited by the chitinase genes in response to invasion of *E. cruciferarum*, *A. brassicicola* and *P. parasitica* are also different in diseased and normal leaves. Compared with PM leaves, three genes (*Bol023322*, *Bol041024* and *Bol035470*) and two genes (*Bol040748* and *Bol021626*) were up- and downregulated, respectively, in normal leaves. Similarly, three genes (*Bol007321*, *Bol023322* and *Bol041024*) and five genes (*Bol035470*, *Bol011420*, *Bol040748*, *Bol021626* and *Bol025197*) were up- and downregulated, respectively, in normal leaves compared to BS leaves. In addition, compared to the leaves with DM, two genes (*Bol041024* and *Bol035470*) and six genes (*Bol010293*, *Bol007321*, *Bol023322*, *Bol011420*, *Bol040748* and *Bol021626*) were up- and downregulated in normal leaves, respectively.

**Fig. 9:**
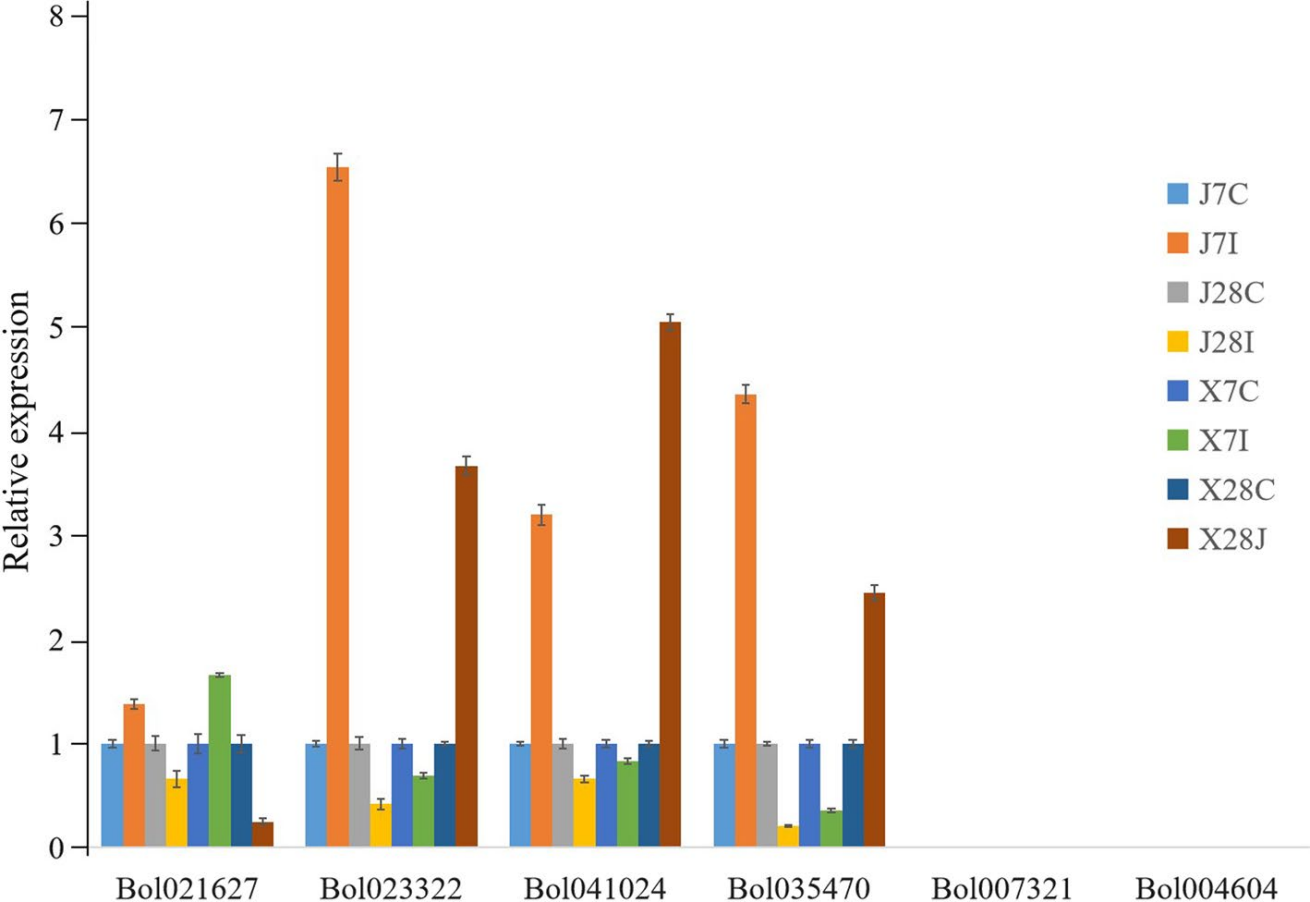
Expression levels of chitinase genes under *P. brassicae* stress. Data are presented as the means ± SD

Under *P. brassicae* infection stress, there was little change in the expression levels of all chitinase genes in the 8 different treatments, which suggesting that chitinase genes played only minor roles in resistance to clubroot*.* The qRT-PCR results for six chitinase genes were also largely consistent with the results of the RNA-seq analysis (Fig. 9).

### Subcellular localization of *Bol040748*

**Fig. 10:**
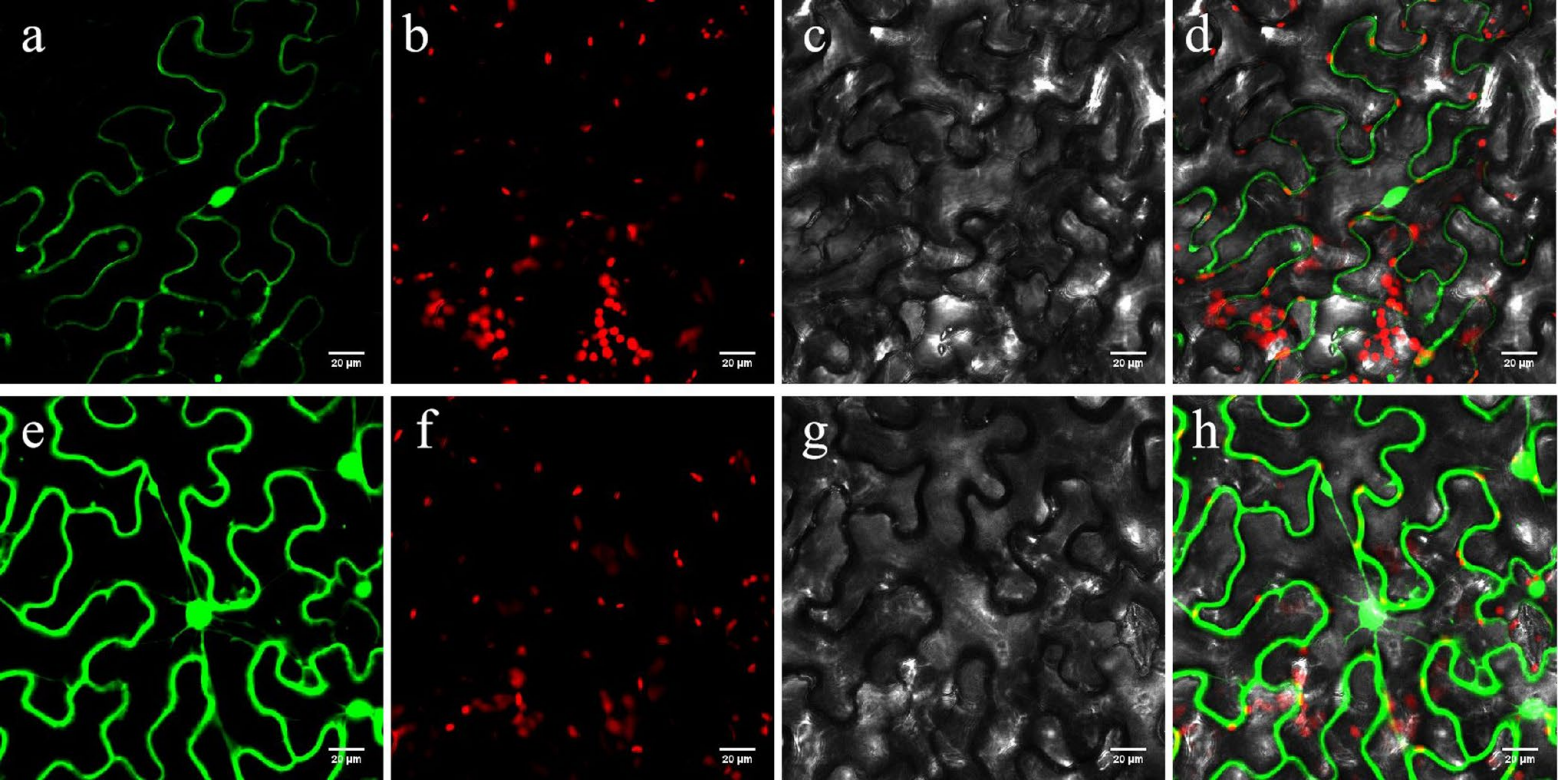
Subcellular localization of *Bol040748* in tobacco protoplast. **a**–**c** and d: Target protein GFP channel, Chloroplast channel, Bright, and Merge. **e**–**h** Control GFP channel, chloroplast channel, bright, and merge. Bars = 20 µm

Protein function was always associated with its subcellular localization. To further understand the protein characteristic of chitinase genes, we recombined the pBWA(V)HS-GLosgfp vector with the CDS sequences of *Bol040748* and then introduced into tobacco protoplasts. As shown in Fig. 10, The GFP signal of *Bol040748* was observed in the cell membrane and nucleus, suggesting that *Bol040748* was a cellular membrane and nuclear protein.

## Discussion

Induced resistance means that the stimulus of the pathogen increases the defense of the plant (Van Loon. 1997). Chitinases, representing a subgroup of pathogenesis-related proteins, play important roles in the plant defense against pathogen invasion (Abeles et al. 1971). Chitinases have been identified in previous studies, and their roles in defending against various pathogens in different crops have been discussed (Kasprzewska. 2003; Rasmussen et al. 1992; Zhou et al. 2017). However, the expression patterns of chitinase genes in response to *F. oxysporum*, *P. brassicae*, *A. brassicicola*, *E. cruciferarum* and *P. parasitica* in cabbage have not been elucidated to date. In this study, 20 chitinase genes were identified, and their phylogenetic relationship, collinearity, structures, chromosomal locations, *cis*-elements and expression patterns in response to the invasion of different pathogens in cabbage were reported. This study provides comprehensive information to characterize the chitinase gene family in cabbage.

Whole genome duplication (WGD) is an important event in plant evolution (Adams et al. 2005). Li et al. (2016) studied the duplicated genes of almost 40 different flowering plants that experienced WGD and found that most of the genes quickly returned to a single-copy status; however, some genes were observed to be consistently present in multiple copies, which belong to gene families that are involved in conditional responses to biotic and abiotic stress and are important for local adaptation (Van de Peer et al. 2017). *Arabidopsis thaliana* has undergone three paleopolyploidy events and two more recent tetraploidy events a and b shared with other members of the order Brassicales (Bowers et al. 2003). In this study, we found that the number of chitinase genes in *B. rapa* (17) and *B. oleracea* (20) nearly doubled or tripled compared with the number in *A. thaliana* (8) (Figs. 1, 3), which suggests that the chitinase genes may be subjected to natural selection pressure and were biased to retain multiple copies after the triploidization event (Cheng et al. 2012).

In this study, we analyzed the expression patterns of 20 chitinase genes under 5 different disease stresses. Specifically, we investigated the chitinase gene members that might play crucial roles in disease resistance. Under FW stress, we detected four genes (*Bol010293*, *Bol007321*, *Bol021626* and *Bol025197*) and five genes (*Bol035464*, *Bol021627*, *Bol035467*, *Bol029469* and *Bol040748*) that were up- and downregulated significantly in 96–100 compared with 01–20. The results of qRT-PCR analysis showed that *Bol004604* and *Bol040748* had completely opposite expression patterns in 96–100 and 01–20, which may indicate that these two genes play an important role in the defense against FW in cabbage. For PM, BS and DM, there were 5, 8 and 8 genes with differential expression in different treatments, respectively. Among these genes, all *Bol023322*, *Bol041024*, *Bol035470*, *Bol040748* and *Bol021626* had different expression levels between diseased and normal leaves under the stress of three diseases, which suggests their pivotal effects on the interaction between cabbage and fungi. In addition, *Bol007321* and *Bol011420* also warranted further study, although they were only differentially expressed between diseased and normal leaves under the stress of BS and DM.

Under the stress of *P. brassicae*, almost all the chitinase genes did not exhibit clear differences in expression, which may suggest that the defense effect of chitinase against clubroot disease was not significant in this study. Coincidentally, from the taxonomic perspective, *P. brassicae* is different from the other four fungi, which may be the reason for the different expression patterns exhibited by the chitinase genes under the pressure of infection by *P. brassicae* and fungi.

## Conclusions

In this study, a genome-wide analysis of *B. oleracea* chitinase genes was performed, and 20 chitinase genes were confirmed. Subsequently, analyses of chitinase genes on gene structures, phylogeny, chromosomal location, gene duplication and gene expression patterns were conducted based on bioinformatic analysis and qRT-PCR. These genes were expressed in various tissues and organs. In addition, there were 9, 5, 8 and 8 genes with differential expression levels in different treatments under the four respective stresses of FW, PM, BS and DM. This study provides comprehensive information for further research investigating the role of chitinase in host plant responses to various diseases.

### *Author contribution statement*

SJL and MZ conceived and designed research. YYZ and HHL conducted experiments. JLJ and XLH collected the public dataset and performed bioinformatics analysis. MZZ wrote the manuscript. ZYF, YW and LMY reviewed the manuscript. All authors read and approved the manuscript.

## Supplementary Information

Below is the link to the electronic supplementary material.

Supplementary file 1 Table S1. The GenBank accession numbers of genes in Arabidopsis. (XLSX 9 KB)

Supplementary file 2 Table S2. Chitinase gene homologs in the genomes of Arabidopsis thaliana and B. oleracea. (XLSX 13 KB)

Supplementary file 3 Table S3. Chitinase homologous genes in the genomes of B. oleracea. (XLSX 11 KB)

Supplementary file 4 Table S4. The primer sequences of 9 chitinase genes used for qRT-PCR. (XLSX 10 KB)

Supplementary file 5 Table S5. Chitinase gene expressions in different organs. (XLSX 11 KB)

Supplementary file 6 Table S6. Chitinase gene expressions in different samples. (XLSX 48 KB)
